# Gynecologic Cancer: Risk Factors, Interception and Prevention

**DOI:** 10.3390/cancers18081235

**Published:** 2026-04-14

**Authors:** Goli Samimi, Brandy Heckman-Stoddard

**Affiliations:** Division of Cancer Prevention, National Cancer Institute, Rockville, MD 20850, USA

Gynecologic cancers account for approximately 100,000 new cases and over 30,000 deaths per year in the United States [1]. Effective treatment of these cancers ranges from local intervention delivery to major surgery, with varying degrees of successful outcomes. Studies have repeatedly demonstrated that early detection and screening, and preventive interventions can improve outcomes by intercepting cancer progression or, in some cases, even inhibiting or prolonging initiation [2]. This Special Issue of *Cancers* presents a series of papers including original research, reviews and meta-analyses, from leaders in the field that cover models, screening and early detection, preventive interventions, risk factors and risk reduction, epidemiology, and health disparities in ovarian, endometrial and cervical cancers. The purpose of this Special Issue is to bring attention to the unique challenges associated with these cancers, and bring to the forefront exciting opportunities for prevention to improve outcomes.

Endometrial, or uterine, cancer is particularly challenging as both incidence and mortality have increased over the past two decades, due in large part to increasing obesity rates in the population, decreasing hysterectomy rates, and an increased proportion of more aggressive subtypes [1]. The timely publication by Clontz et al. undertakes a systematic review and meta-analysis of the association between obesity-related biomarkers and endometrial cancer risk, with the goal of revealing the impact of weight loss on reducing the risk of endometrioid endometrial cancer [3]. The study examined effects of lifestyle interventions, bariatric surgery and pharmacotherapy on obesity and inflammatory biomarkers, and found that despite high heterogeneity limiting statistical significance, weight loss effectively reduces inflammatory and other obesity-related biomarkers associated with increased risk of endometrial cancer.

The Clontz study found that bariatric surgery outperformed both lifestyle and pharmacotherapy interventions; it would be interesting to see how more recent expanded use of glucagon-like peptide-1 (GLP-1) agonists or GLP-1/GIP-1 (gastric inhibitory polypeptide) dual agonists compares to bariatric surgery for sustained weight loss and prevention of endometrial cancer over time. Using disease models, Hagemann et al. explored a combined treatment strategy of semaglutide (a GLP-1 agonist) and progestins for non-surgical management of endometrial cancer [4]. Progestins are widely used hormonal interventions for endometrial cancer prevention, but their effectiveness is limited over time due to high relapse rate. In this study, the authors used endometrial cancer cell lines and patient-derived organoids (PDOs) from grade 1 endometrial cancer to test the hypothesis that combining semaglutide with the progestin levonorgestrel would improve the prevention potential of levonorgestrel alone. Focusing on mechanistic endpoints, the authors found a potential positive feedback loop between semaglutide and the progestin levonorgestrel signaling. As of the time of writing this article, a search for trials investigating GLP-1 agonists in endometrial cancer revealed seven clinical trials in this space. These trials cover GLP-1 agonists in endometrial cancer patients undergoing chemotherapy [5] or following surgical treatment [6] for weight management; GLP-1 agonists in combination with a levonorgestrel intrauterine device (LNG-IUD) in women with endometrioid intraepithelial neoplasia (EIN) or low-grade endometrial cancer for regression or to prevent progression [7,8,9]; and pre-surgical GLP-1 agonists in women with endometrioid intraepithelial neoplasia (EIN) or low-grade endometrial cancer to determine effects on cellular proliferation [10,11]. Mechanistic studies using representative models like the Hagermann study provide the foundation for translation into human studies, and once completed, these trials may reveal additional effects that can then be taken back to the bench for further examination using these models.

Even as these advances help to improve survival in women with endometrial cancer, the undeniable challenge of health disparities persists. Wijayabahu et al. conducted a retrospective cohort study of endometrial cancer cases using the Surveillance, Epidemiology, and End Results (SEER) database to investigate the impact of socioeconomic factors on five-year survival rates of endometrial cancer by race/ethnicity [12]. Survival rates were associated with education, poverty, unemployment and income, with non-Hispanic Black women consistently showing worse survival outcomes compared to other racial/ethnic groups, regardless of socioeconomic characteristics. These findings emphasize the necessity to further explore the persistent endometrial cancer survival disparities for non-Hispanic Black women, as well as the socioeconomic determinants influencing these survival disparities. Any interventional progress made towards improved prevention or outcomes would be dampened without a better understanding of how to apply this improvement towards health equity.

Epithelial ovarian cancer, specifically high-grade serous ovarian cancer (HGSC), faces different challenges in that early detection is rare due to lack of symptoms and ineffective screening options [13], resulting in a majority of cases being diagnosed at a late stage [14]. Clinical management of these patients involves invasive surgery and chemotherapy, with high rates of relapse and high case-fatality rates. These low survival rates in HGSC highlight the importance of early detection and preventive interventions, particularly in women at high risk, as survival rates are markedly improved for women diagnosed at earlier stages [14]. The review by Montemorano et al. presents the current landscape in ovarian cancer prevention, within the framing of the epidemiology of epithelial ovarian cancer as well as our understanding of molecular characteristics and precursor lesions [15]. The authors also describe innovative technological advances in detection and prevention, with the hope that these advances can be eventually applied to future high-risk or potentially population-based screening.

An ideal candidate for effective prevention of any cancer would be both non-invasive and non-toxic. In ovarian cancer, epidemiologic studies have highlighted the potential of oral contraceptives (OCs), which meet both of these criteria, and are strongly associated with a reduced risk [16]. While this link has been repeatedly demonstrated, the specific component(s) of the OCs and the mechanism by which they confer protection is still unknown. Zhai et al. have contributed their preclinical work investigating the various components of OCs using genetically engineered HGSC mouse models [17]. The findings demonstrate that the OC constituent medroxyprogesterone acetate, a progestin, significantly inhibits tumor development and prolongs survival in this mouse model. This publication not only reveals the potential mechanism by which OCs confer protection in HGSC, but also demonstrates the imperativeness of representative models to characterize potential chemo-preventive interventions that would provide non-invasive and non-toxic opportunities for cancer risk reduction.

The current NCCN guidelines for HGSC risk management in high-risk individuals recommend risk-reducing bilateral salpingo-oophorectomy (BSO) before age 40–45 [18]. While this preventive surgery significantly reduces the risk of HGSC, the resulting early menopause increases the risk of osteoporosis, cardiovascular disease and other damaging effects [19]. Research and even some clinical practice has moved towards salpingectomy (with delayed oophorectomy), which offers a preventive option while minimizing adverse events. This approach has been undertaken as opportunistic salpingectomy in some normal-risk populations who are undergoing gynecologic surgery for benign conditions, and has shown promise for HGSC risk reduction [20]. For high-risk individuals, clinical trials are underway to determine whether delayed oophorectomy offers comparable risk reduction compared with risk-reducing BSO [21]. These trials include SOROCk (a non-randomized prospective clinical trial comparing the non-inferiority of salpingectomy to salpingo-oophorectomy to reduce the risk of ovarian cancer among brca1 carriers [22]) and TUBA-WISP-II (tubectomy with delayed oophorectomy as an alternative to risk-reducing salpingo-oophorectomy in high-risk women to assess the safety of prevention [23]). In parallel to these clinical studies, Zhai et al. utilized their HGSC mouse model to compare BSO to salpingectomy to prevent tumor development [24], and found that salpingectomy alone significantly reduced HGSC incidence, while BSO completely prevented tumor development. These preclinical studies provide the opportunity to investigate clinically relevant hypotheses in parallel to definitive clinical trials. These two studies by Zhai et al. demonstrate the value of accurately representative models that reflect not just tumor progression but also reflect precursor stages and tumor development, with adequate latency periods for prevention experiments [17,24].

With ongoing and promising research into preventive interventions for HGSC, the fact remains that identifying high-risk individuals will be imperative for implementing wide-spread prevention programs (surgical or non-surgical) that would positively impact survival outcomes. Contributions by Henrikson et al. [25] and White et al. [26] represent unique approaches towards this challenge. Specifically, they each present a version of Traceback, an approach to identify high-risk families by testing previously diagnosed cases of HGSC who never underwent genetic testing, despite testing guidelines [27]. Both groups utilized clinical data from integrated healthcare registries to identify women previously diagnosed with ovarian cancer. They used different modes of contact for outreach and offering of genetic testing, then applied standard cascade testing approaches to reach family members of those who were found to have a pathogenic or likely pathogenic variant that is associated with HGSC risk. These studies can inform sustainability and implementation of Traceback programs going forward, to increase identification of high-risk families that could be offered safe and effective prevention interventions.

Both papers on cervical cancer highlight expanding opportunities for prevention by improving early detection and enabling less invasive management of precancerous lesions. Khoja et al. emphasize that the global screening guidelines increasingly prioritize HPV-based testing as the most effective strategy for early detection [28]. However, while there is broad agreement on screening ages, inconsistent implementation can lead to a limited impact on mortality and allow disparities to persist, particularly across settings with limited access to vaccination and screening. Hamar et al. demonstrate that topical imiquimod offers a promising conservative option for treating cervical intraepithelial neoplasia (CIN), enhancing HPV clearance and reducing precancerous lesions, while potentially preserving fertility and reducing surgical risks—though conization remains more effective in some cases [29]. Taken together, the findings reinforce that combining accessible HPV vaccination, evidence-based screening, and emerging nonsurgical therapies can strengthen cervical cancer prevention.

Wang et al. contributed an interesting study of the use of tricycle antidepressants (TCAs) and selective serotonin reuptake inhibitors (SSRIs) and association with risk of gynecologic cancers across the board [30]. This epidemiologic study found that use of many TCA and SSRI was significantly associated with lowered risks of cervical, uterine and ovarian cancers, particularly among women aged 40–64, which is also the age range that corresponds with higher risk of these cancer types. Many others have explored repurposing safe interventions for gynecologic cancer prevention, such as metformin as another example [31]. It is essential for additional studies to be undertaken to better understand the potential mechanism for these associations.

Across gynecologic malignancies, the emerging evidence underscores both the urgency and the opportunity to strengthen prevention, early detection, and risk-reduction strategies. Much work remains to expand precision prevention; integrate lifestyle, metabolic, hormonal, and immunologic interventions; advance non-surgical prevention strategies; and integrate emerging technologies, as well as advance implementation work to improve care. Collectively, these directions reflect a rapidly evolving landscape that bridges molecular insights, behavioral science, clinical innovation, and public health implementation—moving toward a future where gynecologic cancer prevention is more precise and more effective across the populations most in need.
